# Neoantigen prioritization based on antigen processing and presentation

**DOI:** 10.3389/fimmu.2024.1487378

**Published:** 2024-11-06

**Authors:** Serina Tokita, Takayuki Kanaseki, Toshihiko Torigoe

**Affiliations:** ^1^ Department of Pathology, Sapporo Medical University, Sapporo, Japan; ^2^ Joint Research Center for Immunoproteogenomics, Sapporo Medical University, Sapporo, Japan

**Keywords:** neoantigen, MHC, antigen processing and presentation, personalized medicine, cancer vaccine

## Abstract

Somatic mutations in tumor cells give rise to mutant proteins, fragments of which are often presented by MHC and serve as neoantigens. Neoantigens are tumor-specific and not expressed in healthy tissues, making them attractive targets for T-cell-based cancer immunotherapy. On the other hand, since most somatic mutations differ from patient to patient, neoantigen-targeted immunotherapy is personalized medicine and requires their identification in each patient. Computational algorithms and machine learning methods have been developed to prioritize neoantigen candidates. In fact, since the number of clinically relevant neoantigens present in a patient is generally limited, this process is like finding a needle in a haystack. Nevertheless, MHC presentation of neoantigens is not random but follows certain rules, and the efficiency of neoantigen detection may be further improved with technological innovations. In this review, we discuss current approaches to the detection of clinically relevant neoantigens, with a focus on antigen processing and presentation.

## Introduction

Mutant peptides derived from somatic mutations are often presented by MHC molecules and give rise to neoantigens (1, 2). Neoantigens are not subject to central tolerance and can therefore induce T cell responses in patients. In preclinical and clinical settings, antitumor effects of immune checkpoint blockade (ICB) are mediated by T cells recognizing neoantigens (3–5). Adoptive cell transfer of neoantigen-reactive CD4^+^ T cells resulted in tumor regression in a patient with epithelial tumor (6). The phase IIb study of the neoantigen vaccine in combination with ICB showed improved recurrence-free survival (RFS) and distant metastasis-free survival in patients with resected melanoma (7). Even in patients with pancreatic cancer, vaccinated patients with neoantigen reactive T cells showed prolonged RFS (8). Vaccination-induced neoantigen-reactive T cells persist for years, suggesting their contribution to long-term prevention of relapse or metastasis (9–11). These observations strongly suggest a central role for neoantigens in mediating host T cell immune responses against tumors, and manipulation of T cell responses to neoantigens may lead to success in cancer immunotherapy (12–15).

Although neoantigens are attractive targets for immunotherapy in a variety of cancers, most (if not all) neoantigens differ between patients. In addition, it is becoming clear that the frequency of immunogenic neoantigens that induce T-cell responses in patients is very low and may be limited to a few percent of somatic mutations (16–19). Thus, neoantigen-targeted immunotherapy becomes personalized medicine, requiring screening for each patient, and such a needle in a haystack must be efficiently identified from a large number of mutations. Advances in next-generation sequencing technology have enabled the identification of nonsynonymous mutations in tumor exomes and the in silico prediction of the affinity between mutant peptides and patient MHC alleles for potential neoantigens. The in silico prediction algorithm for neoantigens is being continuously improved (20, 21). However, when looking at a patient’s T cell response, the percentage of predicted sequences that do not induce a response remains high, making it a challenge to efficiently detect neoantigens by in silico prediction alone (22). In a previous report, neoantigens were predicted using different pipelines from different laboratories, but in total only about 6% of the predicted neoantigens were successfully recognized by the patient’s T cells (23). Such a high false-positive rate may be due to T cell-side factors, such as the diversity of T cell receptor (TCR) repertoires, or tumor cell-side factors, such as the complexity of intracellular antigen processing. In this review, we focus on the latter, highlighting current issues in the identification of clinically relevant neoantigens and potential solutions from an antigen processing perspective with the goal of clinical application of neoantigens.

## What are clinically relevant neoantigens from an antigen presentation perspective?

Naive T cells are primed by professional antigen presenting cells (APCs) in the lymph nodes, and effector T cells subsequently migrate and recognize tumor cells. For a clinically relevant T cell immune response to occur against an antigen, the antigen must be immunogenic (capable of inducing a host T cell response) and presented on cell surfaces (naturally processed and presented by MHC). Although these two requirements are closely related, they are not necessarily the same. For example, in a vaccination setting targeting cytotoxic CD8^+^ T cells, administered neoantigens (or nucleotide sequences encoding neoantigens) will be taken up by APCs and may be able to induce a circulating T cell response in the peripheral blood since they are in principle foreign to the host. However, an anti-tumor effect cannot be expected if the tumor cells themselves do not endogenously present the neoantigens on their MHC. As discussed below, for various reasons, not all expressed gene products with MHC-binding properties are necessarily processed and presented to cell surface MHCs. Conversely, even if tumor cells present immunogenic neoantigens, the neoantigens may fail to elicit a spontaneous endogenous T cell response if cross-presentation of the neoantigens by professional APCs was insufficient. Therefore, clinically relevant neoantigens that elicit spontaneous T cell responses should be those presented by APCs (e.g., cross-presentation) and tumor cells (e.g., endogenous presentation).

In contrast, it is unknown whether this principle applies to MHC-II neoantigens. The significance of MHC-II neoantigen presentation on tumor cells is likely to depend on the phenotype of the neoantigen-reactive CD4^+^ T cells. For helper T cells that exert their antitumor effects indirectly through the production of effector cytokines, by helping to prime CD8^+^ T cells, or by activating myeloid-derived cells, MHC-II neoantigen presentation on tumor cells would not be mandatory, and in fact many tumor cells of epithelial origin lack surface MHC-II expression (24–26). Meanwhile, cytotoxic CD4^+^ T cells capable of lysing tumor cells require MHC-II neoantigen presentation on tumor cells (27, 28). Paradoxically, however, MHC-II neoantigen presentation on tumor cells can also inhibit antitumor effects, possibly by inducing neoantigen-reactive regulatory CD4^+^ T cells in the tumor microenvironment (29). Thus, clinically relevant neoantigens that may lead to therapeutic effects must be presented by MHC; while the case of MHC-I neoantigens is straightforward, the case of MHC-II neoantigens requires further classification and validation with respect to CD4^+^ T cell differentiation types.

## Endogenous antigen processing and MHC class I presentation

**Figure 1:**
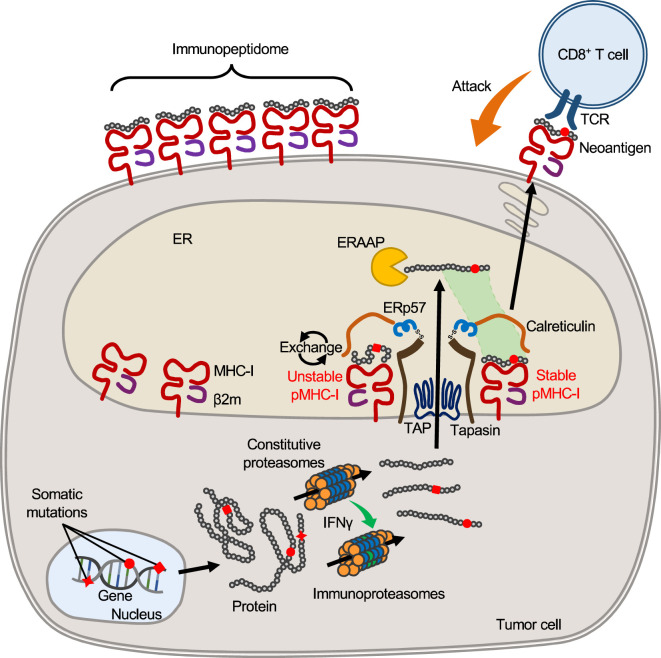
MHC-I antigen processing and presentation pathway. In tumor cells, mutant proteins with substituted amino acids are translated from transcripts with nonsynonymous somatic mutations and, like other proteins, are processed and fragmented by proteasomes in the cytoplasm. Upon IFNγ stimulation, proteasomes are transformed into immunoproteasomes composed of different subunits, yielding protein fragments of different lengths that serve as precursors of MHC-bound peptides. Such peptide precursors are then transported to the ER through the TAP. Inside the ER, an aminopeptidase, ERAAP (ERAP1), trims amino acids from the N-terminus of the precursors, yielding peptides of optimal length for binding to host MHC-I. At the same time, empty MHC-I forms the PLC with β2m, tapasin, calreticulin, and ERp57, which removes and exchanges unstable peptides bound to MHC-I. Stabilized pMHC-I is released from the PLC and transported to the cell surface to form the surface pMHC-I repertoire. When peptides with mutated amino acids are presented, they may serve as neoantigens that elicit a T cell response because they are foreign to the host.

The cell surface repertoire of peptide-MHC-I complex (pMHC-I) is formed by the intracellular antigen presentation pathway consisting of a multi-step process involving the antigen processing machinery (APM) (**Figure 1**) (30–32). The proteasomes degrade proteins in the cytoplasm and yield protein fragments, which serve as precursors of MHC-binding peptides. APCs and tumor cells differ in the composition of proteasome subunits, with APCs expressing the immunoproteasomes and tumor cells of epithelial origin expressing the immunoproteasomes only under IFNγ stimulation. In melanoma patients treated with ICB, higher expression of the immunoproteasomes correlates with improved prognosis, possibly suggesting their influence on neoantigen production with clinical significance (33). The peptide precursors, which are yet too long for MHC binding, are then transported into the endoplasmic reticulum (ER) by the transporters associated with antigen processing (TAP). An ER-resident aminopeptidase, ERAAP (or ERAP1), is present in the ER and trims the amino acids from the N-terminus of the precursors to yield short peptides with optimal lengths for MHC binding (34). Hence, the C-terminal end of peptides is completed in the cytoplasm (by the proteasomes), while the N-terminal end is completed in the ER (by ERAAP). In the endoplasmic reticulum, empty MHC-I molecules associate with TAP, tapasin, ERp57, and calreticulin to form a structure called the peptide loading complex (PLC), which exchanges candidate peptides to form pMHC-I (35). ERAAP and PLC are thought to generate and select peptides that fit into MHC-I molecules, thereby providing stable pMHC-I on the cell surface.

It should be noted that, in theory, cells are not capable of presenting all protein fragments. Since the number of MHC-I molecules (on the order of 10^5^) is limited compared to the number of protein fragments produced in the cytosol, the proportion of intracellular peptides that are ultimately presented by surface MHCs is thought to be very small (36). The actual number of different peptides would be much smaller, since peptides with high gene expression levels are likely to account for multiple copies of MHC-I molecules. Peptides are selected mainly due to their ability to bind to the host MHC-I molecule, but there are other limitations besides MHC binding. For example, ERAAP cannot trim proline, so sequences a few amino acids downstream of proline are unlikely to be presented by MHC-I, even if they have appropriate MHC-I binding motifs (37–39). Thus, not only peptide sequences but also surrounding sequences influence the efficiency of antigen processing. Furthermore, APM expression is not always constant across cells. Loss of APM expression, which is often observed in tumor cells, is likely to influence pMHC-I repertoire formation (40, 41). Thus, the surface MHC-I peptide repertoire is elaborately regulated through endogenous antigen processing pathway; however, the repertoire can be influenced by multiple factors, such as APM expression and competition among candidate peptides, and this complexity may preclude accurate prediction of the peptides presented on the cell surface.

## Landscape of MHC class I presented peptides

Landscapes of peptides displayed by MHC can now be explored by immunopeptidomics or MHC ligandome analysis, in which MHCs are extracted directly from cell lysates and the bound peptides are eluted and comprehensively sequenced by mass spectrometry (MS). The increased sensitivity of MS allows the sequencing of thousands of MHC-I and MHC-II peptides, separately or simultaneously, per sample (42). Sequencing results from a variety of normal and tumor tissue types have been collected and used as training data to improve in silico algorithms for predicting MHC-presented peptides (43). The recovery of naturally presented peptides reveals the nature of antigen processing and presentation. The repertoire of source genes providing an immunopeptidome is not ubiquitous but limited, with only about 60% of expressed protein-coding genes (exon regions) yielding MHC-I represented peptides (44). This proportion is likely to vary further as translation and MHC-I presentation of peptides is now known to occur outside the exons or from unconventional open reading frames (45–47). Furthermore, peptides with higher source gene expression or abundant proteins are more likely to be presented to MHC (38, 48). The distribution of MHC-presented peptide sites within a protein sequence is not also uniform, but rather skewed toward certain sites, often forming “hotspots” (44, 49).Because MHC anchors often contain hydrophobic amino acids, peptide sequences tend to concentrate in transmembrane regions where hydrophobic residues are unevenly distributed (50). The presence of such hotspots clearly indicates that the peptides presented by MHC are not randomly selected from a given protein fragments, but that their selection follows certain biological rules.

## Identification of neoantigens presented by MHC class I or II

As mentioned above, neoantigens with immunogenicity and a higher probability of being presented by MHC need to be prioritized for clinical applications, such as use as vaccines or efficacy biomarkers for ICB. An efficient approach to identify immunogenic neoantigens would be to use the TCRs of clonally expanded tumor-infiltrating T cells (TILs) as a screening probe. Since TILs are already primed and migrating into the tumor, neoantigens recognized by such TCRs should naturally be presented to MHC. High-throughput and sensitive screening platforms have been developed and reported (51–53). Meanwhile, a reliable way to identify MHC-presented peptides would be immunopeptidomics using MS. This approach allows comprehensive identification of MHC-presented neoantigens directly from tissue samples, including solid tumors of epithelial origin (54–57). While this approach may have a lower detection sensitivity due to technical limitations of MS, this approach may be useful for identifying both immunodominant and subdominant neoantigens, the latter of which do not spontaneously induce host T-cell responses and thus may be missed by TIL probes. These MS-detected neoantigens tend to be immunogenic and recognized by patient TILs, potentially suggesting a link between immunogenicity and the level of natural MHC presentation.

**Figure 2:**
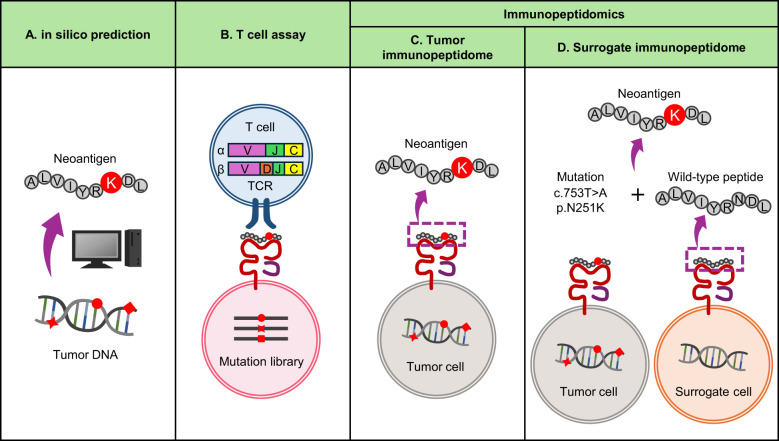
Current approaches to neoantigen identification. **(A)** In silico prediction of neoantigens from genomic data. It is widely used because it can quickly identify neoantigens based on tumor mutation and MHC data. The sensitivity is considered high, but the false positive rate is also high and the specificity is considered low. **(B)** T-cell assay using TIL TCRs as screening probes. A reliable approach to identify immunogenic neoantigens with high specificity, but time consuming and labor intensive. **(C, D)** Immunopeptidomics using MS. A reliable approach to identify neoantigens naturally presented by MHC, but time-consuming and labor-intensive. Sensitivity depends on sample quality and MS performance. **(C)** Conventional immunopeptidomics screening tumor immunopeptidomes. **(D)** Alternative immunopeptidomics screening surrogate (i.e. blood) immunopeptidomes for wild-type counterparts of neoantigens.

On the other hand, both approaches require a specific type of biomaterial, TILs or frozen tumor tissue, which are not always available for every patient in clinical settings and require organized logistics. To overcome this limitation, we propose to exploit the nature of antigen processing and use wild-type surrogate immunopeptidome data instead of tumor immunopeptidomes (**Figure 2**). The majority of immunopeptidomes are matched between different individuals carrying the same MHC type, which may be consistent with the presence of hotspots for MHC presentation in protein sequences.In fact, even among different individuals, about 70% of presenting peptides overlap when MHC types match in the same organ (57, 58). Even between different organs, about 60% of the immunopeptidomes match in both mouse and human, which could be due to the sharing of highly expressed transcripts between different organs (59). Furthermore, MHC presentation of neoantigens is often accompanied by presentation of their wild-type counterparts, as demonstrated by immunopeptidomics (33, 54, 57, 60–63). Neoantigens accompanied with wild-type MHC presentation or those whose wild-type counterparts are already registered in public databases as MHC-presented peptides tend to be immunogenic (64). Therefore, it may be possible to predict naturally presented neoantigens using MHC-matched surrogate wild-type immunopeptidome data in combination with somatic mutation data from the patient’s tumor. We have recently reported an approach called neoantigen selection using surrogate immunopeptidomes (NESSIE), which has efficiently identified immunogenic neoantigens in colorectal and endometrial cancer patients compared to conventional in silico prediction (65). Since this approach analyzes surrogate (e.g. blood) immunopeptidomes but not tumor immunopeptidomes, frozen tumor samples are not required and can be widely used in clinical practice.

In contrast, there are specific types of neoantigens that cannot be detected by NESSIE. As sources of clinically relevant, immunogenic MHC-I neoantigens, SNVs account for 98.2% (56/57) and indels for 1.8% (1/57), with SNV-derived neoantigens being by far the most common (19). Of these, indel-derived neoantigens cannot be detected by NESSIE due to the lack of wild-type counterparts. SNV-derived neoantigens are more complicated. If the mutated amino acid were not an MHC-binding anchor, NESSIE would detect the neoantigen. Even if an MHC-binding anchor was substituted (anchor-type neoantigen), NESSIE would detect the neoantigen unless MHC presentation of its wild-type peptide was completely absent. The percentage of anchor-type neoantigens associated with zero (undetectable) wild-type MHC presentation is unclear. Although there may be limitations as this approach relies on the detection of wild-type counterparts, it is conceptually novel and the sensitivity and specificity of this type of approach compared to conventional methods should be further investigated.

## Conclusion

Neoantigens are truly tumor-specific, a key link between T cells and tumor cells, and thus serve as attractive targets for immunotherapy. However, it is clear that the prioritization of clinically relevant neoantigens remains a challenge and a bottleneck in their clinical application. This challenge would be overcome by technological advances such as the rapid and comprehensive screening of antigens recognized by the TIL repertoire, the development of highly sensitive immunopeptidomics independent of sample quality, and the development of in silico prediction technology based on learning from the results obtained. In particular, although difficult to quantify unambiguously, certain rules exist in the selection of antigens by the antigen processing and presentation pathway, and we consider that their successful application will further improve the efficacy and lead to the success of personalized immunotherapy.

## References

[1] Schumacher TN Schreiber RD. Neoantigens in cancer immunotherapy. Science. (2015) 348:69–74. doi: 10.1126/science.aaa4971 25838375

[2] Schumacher TN Scheper W Kvistborg P. Cancer neoantigens. Annu Rev Immunol. (2019) 37:173–200. doi: 10.1146/annurev-immunol-042617-053402 30550719

[3] Gubin MM Zhang X Schuster H Caron E Ward JP Noguchi T. Checkpoint blockade cancer immunotherapy targets tumour-specific mutant antigens. Nature. (2014) 515:577–81. doi: 10.1038/nature13988 PMC4279952 25428507

[4] Snyder A Makarov V Merghoub T Yuan J Zaretsky JM Desrichard A. Genetic basis for clinical response to CTLA-4 blockade in melanoma. N Engl J Med. (2014) 371:2189–99. doi: 10.1056/NEJMoa1406498 PMC4315319 25409260

[5] Rizvi NA Hellmann MD Snyder A Kvistborg P Makarov V Havel JJ. Cancer immunology. Mutational landscape determines sensitivity to PD-1 blockade in non-small cell lung cancer. Science. (2015) 348:124–8. doi: 10.1126/science.aaa1348 PMC4993154 25765070

[6] Tran E Turcotte S Gros A Robbins PF Lu YC Dudley ME. Cancer immunotherapy based on mutation-specific CD4^+^ T cells in a patient with epithelial cancer. Science. (2014) 344:641–5. doi: 10.1126/science.1251102 PMC6686185 24812403

[7] Weber JS Carlino MS Khattak A Meniawy T Ansstas G Taylor MH. Individualised neoantigen therapy mRNA-4157 (V940) plus pembrolizumab versus pembrolizumab monotherapy in resected melanoma (KEYNOTE-942): a randomised, phase 2b study. Lancet. (2024) 403:632–44. doi: 10.1016/S0140-6736(23)02268-7 38246194

[8] Rojas LA Sethna Z Soares KC Olcese C Pang N Patterson E. Personalized RNA neoantigen vaccines stimulate T cells in pancreatic cancer. Nature. (2023) 618:144–50. doi: 10.1038/s41586-023-06063-y PMC10171177 37165196

[9] Sahin U Derhovanessian E Miller M Kloke BP Simon P Lower M. Personalized RNA mutanome vaccines mobilize poly-specific therapeutic immunity against cancer. Nature. (2017) 547:222–6. doi: 10.1038/nature23003 28678784

[10] Ott PA Hu Z Keskin DB Shukla SA Sun J Bozym DJ. An immunogenic personal neoantigen vaccine for patients with melanoma. Nature. (2017) 547:217–21. doi: 10.1038/nature22991 PMC5577644 28678778

[11] Hu Z Leet DE Allesoe RL Oliveira G Li S Luoma AM. Personal neoantigen vaccines induce persistent memory T cell responses and epitope spreading in patients with melanoma. Nat Med. (2021) 27:515–25. doi: 10.1038/s41591-020-01206-4 PMC8273876 33479501

[12] Sahin U Tureci O. Personalized vaccines for cancer immunotherapy. Science. (2018) 359:1355–60. doi: 10.1126/science.aar7112 29567706

[13] Vormehr M Tureci O Sahin U. Harnessing tumor mutations for truly individualized cancer vaccines. Annu Rev Med. (2019) 70:395–407. doi: 10.1146/annurev-med-042617-101816 30691374

[14] Yamamoto TN Kishton RJ Restifo NP. Developing neoantigen-targeted T cell-based treatments for solid tumors. Nat Med. (2019) 25:1488–99. doi: 10.1038/s41591-019-0596-y 31591590

[15] Blass E Ott PA. Advances in the development of personalized neoantigen-based therapeutic cancer vaccines. Nat Rev Clin Oncol. (2021) 18:215–29. doi: 10.1038/s41571-020-00460-2 PMC7816749 33473220

[16] Tran E Ahmadzadeh M Lu YC Gros A Turcotte S Robbins PF. Immunogenicity of somatic mutations in human gastrointestinal cancers. Science. (2015) 350:1387–90. doi: 10.1126/science.aad1253 PMC7445892 26516200

[17] Linnemann C van Buuren MM Bies L Verdegaal EM Schotte R Calis JJ. High-throughput epitope discovery reveals frequent recognition of neo-antigens by CD4^+^ T cells in human melanoma. Nat Med. (2015) 21:81–5. doi: 10.1038/nm.3773 25531942

[18] Gros A Parkhurst MR Tran E Pasetto A Robbins PF Ilyas S. Prospective identification of neoantigen-specific lymphocytes in the peripheral blood of melanoma patients. Nat Med. (2016) 22:433–8. doi: 10.1038/nm.4051 PMC7446107 26901407

[19] Parkhurst MR Robbins PF Tran E Prickett TD Gartner JJ Jia L. Unique neoantigens arise from somatic mutations in patients with gastrointestinal cancers. Cancer Discovery. (2019) 9:1022–35. doi: 10.1158/2159-8290.CD-18-1494 PMC7138461 31164343

[20] Fotakis G Trajanoski Z Rieder D. Computational cancer neoantigen prediction: current status and recent advances. Immunooncol Technol. (2021) 12:100052. doi: 10.1016/j.iotech.2021.100052 35755950 PMC9216660

[21] Lang F Schrors B Lower M Tureci O Sahin U. Identification of neoantigens for individualized therapeutic cancer vaccines. Nat Rev Drug Discovery. (2022) 21:261–82. doi: 10.1038/s41573-023-00873-5 PMC7612664 35105974

[22] Vitiello A Zanetti M. Neoantigen prediction and the need for validation. Nat Biotechnol. (2017) 35:815–7. doi: 10.1038/nbt.3932 28898209

[23] Wells DK van Buuren MM Dang KK Hubbard-Lucey VM Sheehan KCF Campbell KM. Key parameters of tumor epitope immunogenicity revealed through a consortium approach improve neoantigen prediction. Cell. (2020) 183:818–34.e13. doi: 10.1016/j.cell.2020.09.015 PMC7652061 33038342

[24] Poncette L Bluhm J Blankenstein T. The role of CD4 T cells in rejection of solid tumors. Curr Opin Immunol. (2022) 74:18–24. doi: 10.1016/j.coi.2021.09.005 34619457 PMC8933281

[25] Speiser DE Chijioke O Schaeuble K Munz C. CD4(+) T cells in cancer. Nat Cancer. (2023) 4:317–29. doi: 10.1038/s43018-023-00521-2 36894637

[26] Kruse B Buzzai AC Shridhar N Braun AD Gellert S Knauth K. CD4(+) T cell-induced inflammatory cell death controls immune-evasive tumours. Nature. (2023) 618:1033–40. doi: 10.1038/s41586-023-06199-x PMC10307640 37316667

[27] Oh DY Kwek SS Raju SS Li T McCarthy E Chow E. Intratumoral CD4(+) T cells mediate anti-tumor cytotoxicity in human bladder cancer. Cell. (2020) 181:1612–1625 e1613. doi: 10.1016/j.cell.2020.05.017 32497499 PMC7321885

[28] Cachot A Bilous M Liu YC Li X Saillard M Cenerenti M. Tumor-specific cytolytic CD4 T cells mediate immunity against human cancer. Sci Adv. (2021) 7:eabe3348. doi: 10.1126/sciadv.abe3348 33637530 PMC7909889

[29] Oliveira G Stromhaug K Cieri N Iorgulescu JB Klaeger S Wolff JO. Landscape of helper and regulatory antitumour CD4(+) T cells in melanoma. Nature. (2022) 605:532–8. doi: 10.1038/s41586-022-04682-5 PMC9815755 35508657

[30] Neefjes J Jongsma ML Paul P Bakke O. Towards a systems understanding of MHC class I and MHC class II antigen presentation. Nat Rev Immunol. (2011) 11:823–36. doi: 10.1038/nri3084 22076556

[31] Blum JS Wearsch PA Cresswell P. Pathways of antigen processing. Annu Rev Immunol. (2013) 31:443–73. doi: 10.1146/annurev-immunol-032712-095910 PMC4026165 23298205

[32] Pishesha N Harmand TJ Ploegh HL. A guide to antigen processing and presentation. Nat Rev Immunol. (2022) 22:751–64. doi: 10.1038/s41577-022-00707-2 35418563

[33] Kalaora S Lee JS Barnea E Levy R Greenberg P Alon M. Immunoproteasome expression is associated with better prognosis and response to checkpoint therapies in melanoma. Nat Commun. (2020) 11:896. doi: 10.1038/s41467-020-14639-9 32060274 PMC7021791

[34] Kanaseki T Blanchard N Hammer GE Gonzalez F Shastri N. ERAAP synergizes with MHC class I molecules to make the final cut in the antigenic peptide precursors in the endoplasmic reticulum. Immunity. (2006) 25:795–806. doi: 10.1016/j.immuni.2006.09.012 17088086 PMC2746443

[35] Elliott T Williams A. The optimization of peptide cargo bound to MHC class I molecules by the peptide-loading complex. Immunol Rev. (2005) 207:89–99. doi: 10.1111/j.0105-2896.2005.00311.x 16181329

[36] Rock KL Reits E Neefjes J. Present yourself! By MHC class I and MHC class II molecules. Trends Immunol. (2016) 37:724–37. doi: 10.1016/j.it.2016.08.010 PMC5159193 27614798

[37] Serwold T Gonzalez F Kim J Jacob R Shastri N. ERAAP customizes peptides for MHC class I molecules in the endoplasmic reticulum. Nature. (2002) 419:480–3. doi: 10.1038/nature01074 12368856

[38] Abelin JG Keskin DB Sarkizova S Hartigan CR Zhang W Sidney J. Mass spectrometry profiling of HLA-associated peptidomes in mono-allelic cells enables more accurate epitope prediction. Immunity. (2017) 46:315–26. doi: 10.1016/j.immuni.2017.02.007 PMC5405381 28228285

[39] Hongo A Kanaseki T Tokita S Kochin V Miyamoto S Hashino Y. Upstream position of proline defines peptide-HLA class I repertoire formation and CD8(+) T cell responses. J Immunol. (2019) 202:2849–55. doi: 10.4049/jimmunol.1900029 30936292

[40] Hammer GE Kanaseki T Shastri N. The final touches make perfect the peptide-MHC class I repertoire. Immunity. (2007) 26:397–406. doi: 10.1016/j.immuni.2007.04.003 17459809

[41] Jhunjhunwala S Hammer C Delamarre L. Antigen presentation in cancer: insights into tumour immunogenicity and immune evasion. Nat Rev Cancer. (2021) 21:298–312. doi: 10.1038/s41568-021-00339-z 33750922

[42] Purcell AW Ramarathinam SH Ternette N. Mass spectrometry-based identification of MHC-bound peptides for immunopeptidomics. Nat Protoc. (2019) 14:1687–707. doi: 10.1038/s41596-019-0133-y 31092913

[43] Creech AL Ting YS Goulding SP Sauld JFK Barthelme D Rooney MS. The role of mass spectrometry and proteogenomics in the advancement of HLA epitope prediction. Proteomics. (2018) 18:e1700259. doi: 10.1002/pmic.201700259 29314742 PMC6033110

[44] Pearson H Daouda T Granados DP Durette C Bonneil E Courcelles M. MHC class I-associated peptides derive from selective regions of the human genome. J Clin Invest. (2016) 126:4690–701. doi: 10.1172/JCI88590 PMC5127664 27841757

[45] Laumont CM Daouda T Laverdure JB Bonneil E Caron-Lizotte O Laumont MP. Global proteogenomic analysis of human MHC class I-associated peptides derived from non-canonical reading frames. Nat Commun. (2016) 7:10238. doi: 10.1038/ncomms10238 26728094 PMC4728431

[46] Chong C Muller M Pak H Harnett D Huber F Grun D. Integrated proteogenomic deep sequencing and analytics accurately identify non-canonical peptides in tumor immunopeptidomes. Nat Commun. (2020) 11:1293. doi: 10.1038/s41467-020-14968-9 32157095 PMC7064602

[47] Kikuchi Y Tokita S Hirama T Kochin V Nakatsugawa M Shinkawa T. CD8(+) T-cell immune surveillance against a tumor antigen encoded by the oncogenic long noncoding RNA PVT1. Cancer Immunol Res. (2021) 9:1342–53. doi: 10.1158/2326-6066.CIR-20-0964 34433589

[48] Bassani-Sternberg M Pletscher-Frankild S Jensen LJ Mann M. Mass spectrometry of human leukocyte antigen class I peptidomes reveals strong effects of protein abundance and turnover on antigen presentation. Mol Cell Proteomics. (2015) 14:658–73. doi: 10.1074/mcp.M114.042812 PMC4349985 25576301

[49] Muller M Gfeller D Coukos G Bassani-Sternberg M. 'Hotspots' of antigen presentation revealed by human leukocyte antigen ligandomics for neoantigen prioritization. Front Immunol. (2017) 8:1367. doi: 10.3389/fimmu.2017.01367 29104575 PMC5654951

[50] Bianchi F Textor J. G. van den Bogaart, Transmembrane Helices Are an Overlooked Source of Major Histocompatibility Complex Class I Epitopes. Front Immunol. (2017) 8:1118. doi: 10.3389/fimmu.2017.01118 28959259 PMC5604083

[51] Arnaud M Chiffelle J Genolet R Navarro Rodrigo B Perez MAS Huber F. Sensitive identification of neoantigens and cognate TCRs in human solid tumors. Nat Biotechnol. (2022) 40:656–60. doi: 10.1038/s41587-021-01072-6 PMC9110298 34782741

[52] Cattaneo CM Battaglia T Urbanus J Moravec Z Voogd R de Groot R. Identification of patient-specific CD4(+) and CD8(+) T cell neoantigens through HLA-unbiased genetic screens. Nat Biotechnol. (2023) 41:783–7. doi: 10.1038/s41587-022-01547-0 PMC10264241 36593398

[53] Moravec Z Zhao Y Voogd R Cook DR Kinrot S Capra B. Discovery of tumor-reactive T cell receptors by massively parallel library synthesis and screening. Nat Biotechnol. (2024). doi: 10.1038/s41587-024-02210-6 38653798

[54] Bassani-Sternberg M Braunlein E Klar R Engleitner T Sinitcyn P Audehm S. Direct identification of clinically relevant neoepitopes presented on native human melanoma tissue by mass spectrometry. Nat Commun. (2016) 7:13404. doi: 10.1038/ncomms13404 27869121 PMC5121339

[55] Kalaora S Wolf Y Feferman T Barnea E Greenstein E Reshef D. Combined analysis of antigen presentation and T-cell recognition reveals restricted immune responses in melanoma. Cancer Discovery. (2018) 8:1366–75. doi: 10.1158/2159-8290.CD-17-1418 PMC6453138 30209080

[56] Newey A Griffiths B Michaux J Pak HS Stevenson BJ Woolston A. Immunopeptidomics of colorectal cancer organoids reveals a sparse HLA class I neoantigen landscape and no increase in neoantigens with interferon or MEK-inhibitor treatment. J immunotherapy Cancer. (2019) 7:309. doi: 10.1186/s40425-019-0769-8 PMC6859637 31735170

[57] Hirama T Tokita S Nakatsugawa M Murata K Nannya Y Matsuo K. Proteogenomic identification of an immunogenic HLA class I neoantigen in mismatch repair-deficient colorectal cancer tissue. JCI Insight. (2021) 6:e146356. doi: 10.1172/jci.insight.146356 34185709 PMC8410045

[58] Granados DP Sriranganadane D Daouda T Zieger A Laumont CM Caron-Lizotte O. Impact of genomic polymorphisms on the repertoire of human MHC class I-associated peptides. Nat Commun. (2014) 5:3600. doi: 10.1038/ncomms4600 24714562 PMC3996541

[59] Kubiniok P Marcu A Bichmann L Kuchenbecker L Schuster H Hamelin DJ. Understanding the constitutive presentation of MHC class I immunopeptidomes in primary tissues. iScience. (2022) 25:103768. doi: 10.1016/j.isci.2022.103768 35141507 PMC8810409

[60] Yadav M Jhunjhunwala S Phung QT Lupardus P Tanguay J Bumbaca S. Predicting immunogenic tumour mutations by combining mass spectrometry and exome sequencing. Nature. (2014) 515:572–6. doi: 10.1038/nature14001 25428506

[61] Kochin V Kanaseki T Tokita S Miyamoto S Shionoya Y Kikuchi Y. HLA-A24 ligandome analysis of colon and lung cancer cells identifies a novel cancer-testis antigen and a neoantigen that elicits specific and strong CTL responses. Oncoimmunology. (2017) 6:e1293214. doi: 10.1080/2162402X.2017.1293214 28533942 PMC5433517

[62] Ebrahimi-Nik H Michaux J Corwin WL Keller GL Shcheglova T Pak H. Mass spectrometry driven exploration reveals nuances of neoepitope-driven tumor rejection. JCI Insight. (2019) 5:e129152. doi: 10.1172/jci.insight.129152 31219806 PMC6675551

[63] Minegishi Y Kiyotani K Nemoto K Inoue Y Haga Y Fujii R. Differential ion mobility mass spectrometry in immunopeptidomics identifies neoantigens carrying colorectal cancer driver mutations. Commun Biol. (2022) 5:831. doi: 10.1038/s42003-022-03807-w 35982173 PMC9388627

[64] Muller M Huber F Arnaud M Kraemer AI Altimiras ER Michaux J. Machine learning methods and harmonized datasets improve immunogenic neoantigen prediction. Immunity. (2023) 56:2650-163.e6. doi: 10.1016/j.immuni.2023.09.002 37816353

[65] Tokita S Fusagawa M Matsumoto S Mariya T Umemoto M Hirohashi Y. Identification of immunogenic HLA class I and II neoantigens using surrogate immunopeptidomes. Sci Adv. (2024) 10:eado6491. doi: 10.1126/sciadv.ado6491 39292790 PMC11409964

